# Association of Urinary Sodium Excretion with Vascular Damage: A Local Kidney Effect, Rather Than a Marker of Generalized Vascular Impairment

**DOI:** 10.1155/2018/7620563

**Published:** 2018-12-16

**Authors:** Areti Triantafyllou, Panagiota Anyfanti, Eugenia Gkaliagkousi, Xenophon Zabulis, Anastasios Vamvakis, Vasileios Gkolias, Konstantinos Petidis, Spyros Aslanidis, Stella Douma

**Affiliations:** ^1^3^rd^ Department of Internal Medicine, Papageorgiou Hospital, Aristotle University of Thessaloniki, Thessaloniki, Greece; ^2^Institute of Computer Science, Foundation for Research and Technology – Hellas, Crete, Greece; ^3^2^nd^ Propaedeutic Department of Internal Medicine, Hippokration Hospital, Aristotle University of Thessaloniki, Thessaloniki, Greece

## Abstract

Evidence suggests that increased salt consumption induces blood pressure- (BP) mediated organ damage, yet it remains unclear whether it reflects a generalized micro- and macrovascular malfunction independent of BP. We studied 197 newly diagnosed and never-treated individuals with hypertension, intermediate hypertensive phenotypes, and normal BP, classified by use of 24-hour ambulatory BP monitoring. Sodium excretion and microalbuminuria were estimated in 24-hour urine samples, dermal capillary density was estimated from capillaroscopy, and arterial stiffness was estimated with pulse wave velocity (PWV) and augmentation index (AIx). Sodium excretion correlated with microalbuminuria (*p*<0.001) and 24-hour and day- and nighttime systolic BP, but not with office blood pressure, arterial stiffness, or capillary density. In the multivariate analysis, the association with microalbuminuria was maintained (*p*=0.007). In a population free from the long-standing effects of hypertension, increased salt intake appears to be associated with early signs of vascular kidney damage, rather than a diffuse micro- and macrovascular impairment.

## 1. Introduction

Excessive dietary sodium chloride intake has been acknowledged as a pivotal modifiable environmental risk factor for cardiovascular events that accounts for a rise in blood pressure and promotes hypertension-related target organ damage [1]. Salt consumption has long been listed as a determinant of left ventricular mass and other indices of cardiac structural adaptation to a sustained increase in blood pressure levels [2]. Other studies have demonstrated an association between excessive salt intake and urinary albumin excretion, which represents a well-established prognostic marker for progression to cardiovascular and renal events [3–8].

On the other hand, the impact of salt intake on other vascular beds has not been thoroughly investigated, and only a few data exist regarding the association between salt intake and either arterial stiffness [1, 9, 10] or dermal capillary density [11]. Although the most robust tool for estimating the daily salt intake is the measurement of sodium excretion in 24-hour urine, it has not been uniformly applied [12]. Hence, it remains unanswered whether increased salt intake and thereby enhanced salt excretion exert primarily local damage on the renal capillary network or have a more generalized impact on the vasculature throughout the body, including the micro- and macrocirculation.

Of note, the majority of the previous studies investigating the effects of salt intake on the cardiovascular system have used office blood pressure measurements for the assessment of blood pressure status of the participants. However, 24-hour ambulatory blood pressure monitoring (ABPM) is now being widely used and provides a reliable prognostic index in terms of cardiovascular morbidity and mortality, considered by many as superior to office blood pressure recordings [13]. It is thus important to determine whether salt intake correlates with specific 24-hour ABPM parameters, for example, daytime and nighttime blood pressure measurements or dipping status. Moreover, ABPM can offer further and accurate classification into true hypertensives, normotensives, and patients with intermediate hypertension phenotypes (white-coat and masked hypertensives) with the last ones bearing an intermediate cardiovascular risk [14]. It is unknown whether salt consumption varies among different hypertension phenotypes.

The primary aim of the present study was to investigate whether an association exists between urinary sodium excretion and different forms of subclinical micro- and macrovascular organ damage (microalbuminuria in the kidney, capillary rarefaction of the skin, and arterial stiffness of the large vessels) independent of blood pressure levels, at the same time and long before the establishment of overt cardiovascular or renal disease. We additionally investigated whether increased salt excretion (i) correlates with 24-hour ABPM parameters and (ii) is related to blood pressure status, classified by use of ABPM, in a naïve population of newly diagnosed individuals, never-treated individuals, otherwise healthy patients with hypertension, and intermediate hypertensive phenotypes, as well as normotensive individuals.

## 2. Methods

### 2.1. Participants

The study population consisted of consecutive patients who attended our Hypertension Unit and normotensive, healthy individuals admitted for regular check-up. All participants were Caucasians and gave written informed consent. The study was approved by the Ethics Committee of Aristotle University of Thessaloniki and was conducted in accordance with the principles of the Helsinki Declaration. In order to participate, all individuals had to not have been previously treated with antihypertensive agents, had to not receive medication of any kind, and had to be free of any other known health problems. History, medical examination, and laboratory tests were used as indicated to verify absence of secondary causes of hypertension and other comorbidities (including diabetes mellitus and cardiovascular diseases). Alcohol and smoking habits were recorded and body mass index (BMI) was calculated as weight (kg)/height^2^ (m^2^).

### 2.2. Blood Pressure Measurements

Using standard methodology, office blood pressure (ofBP) was measured after 10 minutes at rest and was determined as the mean of the second and third value of three consecutive BP recordings taken at a 2-minute interval. A Spacelabs 90207 device was applied to all participants to obtain 24-hour ABPM recordings, which were performed every 20 minutes during a usual working day and every 30 minutes during the night. Successful readings should account for ≥70% in order to be considered technically sufficient. A dipping pattern was defined as a difference ≥10% between the daytime and nighttime systolic BP (SBP). Office and ABPM recordings were combined to classify participants into the following groups: patients with true hypertension (TH) (ofBP≥140/90 mmHg and daytime ABPM≥135/85 mmHg), patients with intermediate hypertensive phenotypes (i.e., masked hypertensives (MH), those with ofBP <140/90 mmHg and daytime ABPM≥135/85 mmHg, and white-coat hypertensives (WCH), those with ofBP≥140/90 mmHg and daytime ABPM<135/85 mmHg)), and normotensive individuals (NT) (ofBP<140/90 mmHg and daytime ABPM<135/85 mmHg).

### 2.3. Biochemical Measurements

Blood samples were derived from all patients in the morning after overnight fasting, with patients lying in the supine position for two hours before blood sampling. Lipids and kidney function were estimated from serology, while plasma renin activity (PRA) (ng/mL/h) and serum aldosterone (ng/dl) levels were estimated with the radioimmunoassay method. Glomelural Filtration Rate (GFR) estimation was based on the Cockcroft-Gault formula.

24-hour urine samples were collected for the measurement of urinary sodium and albumin excretion. The latter was measured using the immunoturbidimetric method. Estimation of microalbuminuria, defined as urinary albumin excretion (UAE) rate between 30 and 300 mg/24h, with the above method in 24-hour urine samples is considered the most reliable method of microalbuminuria estimation [15]. Likewise, sodium excretion in 24-hour urine samples is considered more reliable compared to random or consecutive urine samples for daily sodium intake estimation [16]. Participants were advised against any change in their usual dietary habits.

### 2.4. Capillaroscopy Photography and Analysis

Nailfold capillaroscopy (DS Medica, Milan, Italy, 200 x magnification) was applied to all participants. Semiautomated software was developed by our Hypertension Unit and FORTH (Foundation for Research and Technology-Hellas) for the detection and measurement of the number of capillaries in each obtained image. A grader masked to the subjects' identity and BP group assignment examined the microscopic images of each participant and chose a pair of them, according to their quality. A trained operator analyzed semiautomatically at least two measurements from each participant. Capillary rarefaction was defined as the lower tertile of a population consisting of 250 otherwise healthy, except for the high blood pressure, individuals. The intraclass correlation coefficient for a set of 20 patients was 0.951 (with confidence intervals 95%, 0.859-0.983). The applied software and protocol have been analyzed elsewhere in detail [17, 18].

### 2.5. Estimation of Aortic Stiffness

The SphygmoCor device (AtCor Medical, Sydney, Australia) was used to assess arterial stiffness by measurement of pulse wave velocity (PWV), according to a standard protocol. After a 15-minute resting period in the supine position, waveforms at the right common carotid and right femoral site were recorded sequentially. Surface distance between the two recording sites was measured (sternal notch to carotid site and sternal notch to femoral site). Wave transit time was calculated using a simultaneously recorded electrocardiogram as a reference.

The aortic augmentation index (AIx) was expressed as a percentage of the ratio of augmentation pressure to central pulse pressure (the difference between central systolic and diastolic pressure). For the present analyses, we used AIx corrected for the mean (75) heart rate (AIx@75).

### 2.6. Statistical Analysis

Analysis was performed using the Statistical Package for Social Sciences (SPSS) 24. Differences between mean values for continuous variables were estimated by the analysis of variance (ANOVA) and with independent samples Kruskal-Wallis test for the parameters with normal and nonnormal distribution, respectively. A Bonferroni correction was used where necessary for making all eligible pairwise comparisons. Qualitative variables were compared by the chi-squared test. Continuous variables were described as mean ± standard deviation (SD) or as median (interquartile range) according to the normality of their distribution. Correlation coefficients were calculated with Pearson's rank test and the partial correlation coefficient after adjusting for age, BMI, and smoking. To explore if the relationship between sodium excretion and microalbuminuria remained after controlling for other covariates, we applied models of multivariate linear regression analysis. Where needing to transform a nonnormal to a normal distribution, we used the logarithmic mean of the parameter. A probability value of* p*≤0.05 was considered statistically significant.

## 3. Results

A total of 197 individuals with a mean age of 43.7 ± 12.1 years were studied: 109 true hypertensives, 43 with intermediate hypertension phenotypes, and 45 normotensive individuals. Baseline characteristics of the above groups are depicted in Table 1. Sodium excretion was similar between TH, individuals with intermediate hypertension phenotypes, and NT individuals (Table 1). By contrast, aldosterone levels (*p*=0.034), PWV (*p*<0.001), BMI (*p*=0.029), and UAE (*p*=0.039) were significantly higher among ΤΗ compared to NΤ, whereas capillary rarefaction, though more pronounced in TH compared to NT (37% vs. 24%), did not significantly differ between the groups.

We further investigated whether any associations existed between sodium excretion and indices of micro- and macrovascular function. In the univariate analysis, sodium excretion significantly correlated with UAE (r=0.302,* p*<0.001), but not with AIx (*p*=0.096), PWV (*p*=0.895), or capillary density (*p*=0.206). In addition, sodium excretion correlated with BMI (r=0.286,* p*<0.001) and HDL (r=-0.150,* p*=0.042), but no significant associations were observed with age (*p*=0.755), total cholesterol (*p*=0.422), PRA (*p*=0.116), or aldosterone levels (*p*=0.608).

When we assessed the relationship between sodium excretion and office and ambulatory BP parameters, we observed that sodium excretion was significantly associated with 24-hour SBP (*p*<0.01), daytime SBP (*p*<0.01), and nighttime SBP (*p*<0.01), but not with office blood pressure measurements (Table 2). After adjustment for other factors (age, BMI, and smoking), the association between sodium excretion and 24-hour SBP (*p*<0.05), as well as nighttime SBP (*p*<0.05) and daytime SBP (*p*<0.05), remained statistically significant (Table 2).

Further classification of our population according to their dipping status showed that sodium excretion was not statistically different between dippers and nondippers (153.9 (96.5-224.1) vs. 135.2 (83.25-183.6) mmol/24 hours,* p*=0.097). On the contrary, UAE was significantly increased in nondippers as compared to dippers (25.5 (13.3-45.2) vs. 15.3 (7.3-29) mg/24 hours,* p*=0.001).

To verify whether the above associations remained statistically significant independently of other covariables, we developed a multivariable linear regression model (Table 3). Only the association between sodium excretion and UAE (*p*=0.007) remained significant in the multivariate regression model.

## 4. Discussion

A large body of evidence suggests that sodium intake in excess of physiological needs represents an essential contributor to hypertension and mediates cardiovascular and renal dysfunction. In the present study, we investigated whether an association exists between sodium excretion and indices of micro- and macrovascular function independent of blood pressure levels, in a population including all blood pressure phenotypes (true hypertensives, intermediate hypertensive phenotypes, and normotensive individuals). Of the microvascular indices that were applied, urinary sodium excretion significantly correlated with microalbuminuria in our population. This result is consistent with findings from previous studies [4–8] and in accordance with the current knowledge in the field [7]. On the contrary, a nonsignificant association was observed with capillary rarefaction. Even though it appears that nailfold capillary density might have a role in cardiovascular risk prediction in hypertension [19], there is just one previous study [11] dealing with capillary density and 24-hour urinary sodium in 169 multiethnic hypertensive patients, which showed not only a significant inverse association, independent of blood pressure levels, but also an improvement in dermal capillary rarefaction after salt intake reduction. However, a limitation of this study is that only hypertensive patients of a heterogeneous racial origin were studied. By contrast, our findings suggest that, in a population consisting of not only TH and NT but also masked and white-coat hypertensive individuals, increased sodium urine excretion primarily exerts a local effect on the microcirculation of the renal glomerulus, rather than a generalized microvascular dysfunction (evidenced by dermal capillary density). Of note all subjects were free from long-term cardiovascular and renal effects of essential hypertension.

Of the macrovascular markers that were studied (AIx, PWV), none was associated with sodium excretion. Previous studies focusing on the association between salt intake and arterial stiffness are rare and there is still no consolidation regarding whether salt excretion exerts any effects independent of BP on the large artery walls. A previous study of 49 community-dwelling individuals failed to demonstrate a relationship between 24-hour sodium excretion and brachial-ankle PWV. However, the study was methodologically limited and the sample was relatively small [1]. The largest study comes from Liu et al., who randomly recruited 630 participants from a Flemish general population. Sodium excretion was measured at baseline and at a median follow-up of 9.7 years, while AIx was measured at follow-up only. In multivariable-adjusted longitudinal analyses, a 40 mmol/L (~1 SD) increase in the urinary sodium concentration was independently and inversely associated with the aortic AIx. In cross-sectional analyses of follow-up data, these estimates remained statistically significant, but, in the longitudinal and cross-sectional analyses, AIx was unrelated to the 24-hour urinary excretion of sodium [9].

Another important finding of our study is the association between sodium intake and 24-hour ABPM parameters. We showed for the first time that urinary sodium excretion significantly correlated with 24-hour and day- and nighttime SBP, even after adjustment for other parameters, in a meticulously selected sample of 197 normotensive and newly diagnosed hypertensive individuals. A significant positive correlation between ambulatory mean blood pressure and urinary sodium excretion has been previously found in a study of 12 normotensive individuals, conducted by Centonza et al. [20]. Previous reports demonstrating a positive association between ambulatory nocturnal blood pressure and urinary excretion have focused on individuals with normal BP levels [20, 21] or recruited individuals from the community (301 male London civil servants) [22]. In the latter study, no correlation was found between sodium excretion and daytime or 24-hour blood pressure, leading the authors to the conclusion that overnight urinary sodium could at least partially reflect pressure diuresis and thus cannot be employed to assess the association between salt intake and blood pressure [22]. Likewise, in the studies by Centonza et al. and Staessen et al., it was proposed that the association between salt excretion and nighttime blood pressure was mediated by humoral factors and particularly the variation of aldosterone over day and night [20, 21]. While no association was demonstrated between aldosterone or PRA levels and sodium excretion in our study, both 24-hour and day- and nighttime SBP correlate with sodium excretion (Table 2). Our results suggest that the amount of daily sodium intake affects not only the daytime but also nocturnal blood pressure, while this effect does not appear to be mediated by aldosterone levels.

Regarding nighttime dipping, sodium excretion in our population did not differ according to dipping status. This result is consistent with a recent study which reported similar levels of sodium excretion regardless the dipping category among 269 hypertensive patients [23], in spite of the multiple comorbidities and the polypharmacy of the population [24]. By contrast, UAE was significantly increased among nondippers, compared to dippers, in our study. There is still no consistency in the literature regarding UAE levels according to the dipping status, with some studies showing increased [23, 25] and others similar levels of UAE [26, 27] among nondippers, compared to dippers. Taking into account the well-acknowledged prognostic value of microalbuminuria in terms of cardiovascular mortality and morbidity, our findings are in accordance with the wealth of evidence demonstrating an aggravated cardiovascular profile of nondippers [28].

Interestingly, an association of urinary sodium excretion was not observed with office blood pressure levels in our study. Although the jury is still out on whether ABPM represents a more accurate prognostic index in terms of cardiovascular morbidity, mortality, and target organ damage, compared to office blood pressure measurements [13], our findings suggest that 24-hour ABPM is a more sensitive indicator of 24-hour sodium excretion compared to conventional office blood pressure recordings, at least in a well-defined population free from the long-term effects of essential hypertension. However, the fact that while sodium excretion in 24-hour urine samples is the gold standard measure of sodium intake, in comparison to salt intake questionnaires and sodium excretion measurement in overnight samples, it may still present great variability according to the dietary habits within the day [12, 29, 30] needs to be taken into account.

Finally, the fact that 24-hour urinary sodium excretion can change rapidly and sodium intake effect on blood pressure can be evident within a relatively short time frame needs to be remembered [31]; the changes of micro- and macrocirculation measurements may need time to happen.

## 5. Conclusions

In the present study, we showed that UAE, but not capillary rarefaction of the skin or arterial stiffness, correlated with increased sodium excretion independently of blood pressure levels, for the first time in an untreated, free-from-other-comorbidities population of different blood pressure phenotypes. Our findings suggest that increased salt excretion primarily affects the kidney and it is probably not accompanied by pronounced vascular dysfunction in other vascular beds, in a population free from the long-term effects of hypertension. In addition, we showed that even though salt excretion was comparable between the study subgroups, it was significantly associated with both 24-hour and day- and nighttime SBP, but not with office blood pressure, suggesting that dietary sodium intake might be more accurately reflected in ambulatory rather than conventional office blood pressure recordings. Hence, modification of salt intake could influence cardiovascular risk through effects on the renal microvasculature and blood pressure variations over the whole day- and nighttime period.

## Figures and Tables

**Table 1:** Baseline characteristics of the study population.

	**TH**	**IHP**	**NT**	***p* value**
	(n=109)	(n=43)	(n=45)	
**Age, years **	43.7 ± 11.8	44.2 ± 13.5	42.8 ± 11.8	0.875
**BMI, Kg/m** ^**2**^	27.7 ± 4.3^#^	27.5 ± 3.9	25.8 ± 4.1	0.029
**Sex (male, **%**)**	65.4	61.4	49.1	0.058
**Smoking (**%**)**	45.6	31.6	32.7	0.061
**SBP, mmHg**	151.6 ± 14.0^*∗*^	136.4 ± 13.7^*∗*^	118.3 ± 9.0	<0.001
**DBP, mmHg**	94.8 ± 10.2^*∗*^	85.9 ± 8.6^*∗*^	73.2 ± 8.5	<0.001
**24h SBP, mmHg**	141.1 ± 11.1^*∗*^	127.6 ± 8.7^*∗*^	115.8 ± 7.4	<0.001
**24h DBP, mmHg**	88.9 ± 9.4^*∗*^	80.2 ± 6.5^*∗*^	73.2 ± 5.4	<0.001
**Day SBP, mmHg**	146.7 ± 10.9^*∗*^	133.2 ± 9.4^*∗*^	120.3 ± 7.3	<0.001
**Day DBP, mmHg**	93.1 ± 9.2^*∗*^	84.7 ± 6.8^*∗*^	77.5 ± 5.4	<0.001
**Night SBP, mmHg**	128.6 ± 13.8^*∗*^	115.2 ± 9.3^*∗*^	104.8 ± 8.6	<0.001
**Night DBP, mmHg**	78.9 ± 11.4^*∗*^	69.6 ± 7.3	64.8 ± 7.1	<0.001
**Dippers (**%**)**	65.7	73.7	66.7	0.705
**Total cholesterol (mg/dl)**	202.1 ± 40.4	204.1 ± 44.7	193.2 ± 36.5	0.386
**GFR (ml/min/1.73m** ^**2**^ **)**	118.7 ± 34.5	110.7 ± 27.8	110.0 ± 32.0	0.216
**PRA (ng/ml/h)**	0.39 (0.13-1.21)	0.70 (0.20-1.33)	0.69 (0.16-1.55)	0.444
**Aldosterone (ng/100ml)**	10.8 (7.1-16.3)^#^	10.1 (6.3-16.1)	7.5 (5.4-11.7)	0.034
**24h urine sodium (mmol/24h)**	145.6(97.9-199.8)	136(82.7-187.5)	118.8(70.9-187.5)	0.255
**Augmentation index 75**%	23.2 ± 13.0	20.5 ± 10.5	18.5 ± 17.1	0.229
**PWV (m/sec)**	8.08(7.2-9.3)^*∗*^	7.8(7.2-8.6)^*∗*^	6.8(6.1-7.4)	<0.001
**24h urine albumin (mg/24h)**	19.2 (10.0-33.0)^#^	24.0 (12.0-35.0)^@^	11.7 (5.8-27.6)	0.039
**Capillary rarefaction (**%**)**	37.0	30.8	24.0	0.120

TH: true hypertensives, IHP: intermediate hypertensive phenotypes, and NT: normotensives.

SBP: systolic blood pressure, DBP: diastolic blood pressure, MBP: mean blood pressure, BMI: body mass index, GFR: Glomerular Filtration Rate, PRA: plasma renin activity, h: hour, and PWV: pulse wave velocity.

Continuous variables were described as mean ± standard deviation (SD) or as median (interquartile range) according to the normality of their distribution.

Comparisons among qualitative variables were made by chi-squared analysis and among quantitative variables with the analysis of variance (ANOVA) with the Bonferroni adjustment for multiple comparisons.

*∗* : *p*≤0.001, $: *p*<0.01, #: *p*<0.05, and @: *p*=0.059 for differences between normotensives and the other groups.

**Table 2:** Correlation of sodium excretion with office and ambulatory blood pressure components. Without and after adjustment for age, BMI, and smoking.

	**Office BP**		**Daytime**		**Nighttime**		**24-hour**	
	**SBP**	**DBP**	**SBP**	**DBP**	**SBP**	**DBP**	**SBP**	**DBP**
Sodium excretion mmol/24h	0.118	0.024	0.214*∗∗*	0.114	0.219*∗∗*	0.142	0.207*∗∗*	0.125
Adjusted r^+^	0.113	-0.470	0.188*∗*	0.075	0.186*∗*	0.077	0.208*∗*	0.096

*∗p*<0.05, *∗∗p*<0.01.

SBP: systolic blood pressure, DBP: diastolic blood pressure, ΒMI: body mass index, and h: hour.

^+^ r adjusted (partial correlation) for age, BMI, and smoking.

**Table 3:** Multiple linear regression models for factors predicting urinary sodium excretion 24h urine sodium (mmol/24h) (adjusted R square=0.168, R square=0.209, *p*<0.001).

	**Unstandardized**	**LB**	**UB**	**Standardized**	***p***
	**coefficient**			**coefficient**	
**Age (years)**	0.003	-0.002	0.007	0.149	0.210
**Sex (Male**%**)**	-0.055	-0.155	0.046	-0.112	0.285
**BMI (Kg/m** ^**2**^ **)**	0.010	-0.001	0.022	0.173	0.087
**Night SBP (mmHg)**	0.003	0.000	0.006	0.188	0.068
**Dipping**	0.003	-0.004	0.010	0.085	0.382
**HDL, mg/dl**	0.001	-0.003	0.004	0.043	0.626
**GFR **	0.000	-0.001	0.002	0.026	0.799
**24h urine albumin (mg/24h)**	0.119	0.033	0.204	0.235	0.007
**AIx@75**%	-0.004	-0.007	0.000	-0.211	0.072
**Capillaries (number/mm** ^**3**^ **)**	0.000	-0.001	0.002	0.031	0.719

ΒMI: body mass index, SBP: systolic blood pressure, AIx: augmentation index, CI: confidence intervals, LB: lower boundary, and UB: upper boundary.

## Data Availability

All data as well as original medical datasets of all the patients exist in the Internal Medicine Clinic of Papageorgiou Hospital.

## References

[1] Du Cailar G., Ribstein J., Mimran A. (2002). Dietary sodium and target organ damage in essential hypertension. *American Journal of Hypertension*.

[2] Schmieder R. E., Messerli F. H., Garavaglia G. E., Nunez B. D. (1988). Dietary salt intake: A determinant of cardiac involvement in essential hypertension. *Circulation*.

[3] Polonia J., Monteiro J., Almeida J., Silva J. A., Bertoquini S. (2016). High salt intake is associated with a higher risk of cardiovascular events: A 7.2-year evaluation of a cohort of hypertensive patients. *Blood Pressure Monitoring*.

[4] Poulter N. (1998). Dietary sodium intake and mortality: NHANES. The Faculty 31st International Society and Federation of Cardiology 10-day Teaching Seminar in Cardiovascular Disease, Epidemiology and Prevention. *The Lancet*.

[5] He J., Ogden L. G., Vupputuri S., Bazzano L. A., Loria C., Whelton P. K. (1999). Dietary sodium intake and subsequent risk of cardiovascular disease in overweight adults. *Journal of the American Medical Association*.

[6] Tunstall-Pedoe H., Woodward M., Tavendale R., A' Brook R., McCluskey M. K. (1997). Comparison of the prediction by 27 different factors of coronary heart disease and death in men and women of the Scottish heart health study: Cohort study. *British Medical Journal*.

[7] Khaledifar A., Gharipour M., Bahonar A., Sarrafzadegan N., Khosravi A. (2013). Association between salt intake and albuminuria in normotensive and hypertensive individuals. *International Journal of Hypertension*.

[8] de Zeeuw D., Remuzzi G., Parving H.-H. (2004). Albuminuria, a therapeutic target for cardiovascular protection in type 2 diabetic patients with nephropathy. *Circulation*.

[9] Liu Y.-P., Thijs L., Kuznetsova T. (2013). Central systolic augmentation indexes and urinary sodium in a white population. *American Journal of Hypertension*.

[10] Redelinghuys M., Norton G. R., Scott L. (2010). Relationship between urinary salt excretion and pulse pressure and central aortic hemodynamics independent of steady state pressure in the general population. *Hypertension*.

[11] He F. J., Marciniak M., Markandu N. D., Antonios T. F., MacGregor G. A. (2010). Effect of modest salt reduction on skin capillary rarefaction in white, black, and Asian individuals with mild hypertension. *Hypertension*.

[12] Wang C.-Y., Cogswell M., Loria C. (2013). Urinary excretion of sodium, Potassium, and Chloride, but not Iodine, varies by timing of collection in a 24-hour calibration study. *Journal of Nutrition*.

[13] Dolan E., Stanton A., Thijs L. (2005). Superiority of ambulatory over clinic blood pressure measurement in predicting mortality: The Dublin outcome study. *Hypertension*.

[14] Mancia G., Fagard R., Narkiewicz K. (2013). 2013 ESH/ESC Guidelines for the management of arterial hypertension: the Task Force for the management of arterial hypertension of the European Society of Hypertension (ESH) and of the European Society of Cardiology (ESC). *Journal of Hypertension*.

[15] American Diabetes Association (2000). Diabetic nephropathy. *Diabetes Care*.

[16] O'Donnell M., Mente A., Yusuf S. (2015). Sodium Intake and Cardiovascular Health. *Circulation Research*.

[17] Triantafyllou A., Anyfanti P., Zabulis X. (2014). Accumulation of microvascular target organ damage in newly diagnosed hypertensive patients. *Journal of the American Society of Hypertension*.

[18] Karamaounas P., Zabulis X. (2011). *Capillaroscope*.

[19] Triantafyllou A., Anyfanti P., Pyrpasopoulou A., Triantafyllou G., Aslanidis S., Douma S. (2015). Capillary Rarefaction as an Index for the Microvascular Assessment of Hypertensive Patients. *Current Hypertension Reports*.

[20] Centonza L., Castoldi G., Chianca R. (2000). Short-term analysis of the relationship between blood pressure and urinary sodium excretion in normotensive subjects. *Clinical Science*.

[21] Staessen J. A., Birkenhäger W., Bulpitt C. J. (1993). The relationship between blood pressure and sodium and potassium excretion during the day and at night. *Journal of Hypertension*.

[22] Staessen J., Broughton P. M. G., Fletcher A. E. (1991). The assessment of the relationship between blood pressure and sodium intake using whole-day, daytime and overnight urine collections. *Journal of Hypertension*.

[23] Afsar B., Elsurer R. (2010). Urinary albumin excretion among nondipper hypertensive patients is closely related with the pattern of nondipping. *Journal of the American Society of Hypertension*.

[24] Anyfanti P., Triantafyllou A., Douma S. (2015). Hemodynamic and Arterial Stiffness Parameters in Ambulatory Blood Pressure Phenotypes and the Clinical Scenario of Polypharmacy and Comorbidities. *The Journal of Clinical Hypertension*.

[25] Bianchi S., Bigazzi R., Baldari G., Sgherri G., Campese V. M. (1994). Diurnal variations of blood pressure and microalbuminuria in essential hypertension. *American Journal of Hypertension*.

[26] Cuspidi C., Sala C., Valerio C., Negri F., Mancia G. (2012). Nocturnal hypertension and organ damage in dippers and nondippers. *American Journal of Hypertension*.

[27] Cuspidi C., Meani S., Salerno M. (2004). Cardiovascular target organ damage in essential hypertensives with or without reproducible nocturnal fall in blood pressure. *Journal of Hypertension*.

[28] Hansen T. W., Li Y., Boggia J., Thijs L., Richart T., Staessen J. A. (2011). Predictive role of the nighttime blood pressure. *Hypertension*.

[29] Polonia J., Martins L., Pinto F., Nazare J. (2014). Prevalence, awareness, treatment and control of hypertension and salt intake in Portugal: Changes over a decade the PHYSA study. *Journal of Hypertension*.

[30] Yongqing Z., Ming W., Jian S. (2016). Prevalence, awareness, treatment and control of hypertension and sodium intake in Jiangsu Province, China: A baseline study in 2014. *BMC Public Health*.

[31] Meneton P., Jeunemaitre X., de Wardener H. E., Macgregor G. A. (2005). Links between dietary salt intake, renal salt handling, blood pressure, and cardiovascular diseases. *Physiological Reviews*.

